# Silver Nanoparticles Inhibit Metastasis of 4T1 Tumor in Mice after Intragastric but Not Intravenous Administration

**DOI:** 10.3390/ma15113837

**Published:** 2022-05-27

**Authors:** Kamil Brzóska, Maria Wojewódzka, Małgorzata Szczygiel, Agnieszka Drzał, Martyna Sniegocka, Dominika Michalczyk-Wetula, Eva Biela, Martyna Elas, Małgorzata Kucińska, Hanna Piotrowska-Kempisty, Lucyna Kapka-Skrzypczak, Marek Murias, Krystyna Urbańska, Marcin Kruszewski

**Affiliations:** 1 Centre for Radiobiology and Biological Dosimetry, Institute of Nuclear Chemistry and Technology, Dorodna 16, 03-195 Warsaw, Poland; m.wojewodzka@ichtj.waw.pl (M.W.); m.kruszewski@ichtj.waw.pl (M.K.); 2 Department of Biophysics and Cancer Biology, Faculty of Biochemistry, Biophysics and Biotechnology, Jagiellonian University, Gronostajowa 7, 30-387 Kraków, Poland; gosia.szczygiel@uj.edu.pl (M.S.); agnieszka.drzal@uj.edu.pl (A.D.); martyna.sniegocka@gmail.com (M.S.); dominika.michalczyk@uj.edu.pl (D.M.-W.); ewka.biela@gmail.com (E.B.); martyna.elas@uj.edu.pl (M.E.); krystyna.urbanska@uj.edu.pl (K.U.); 3 Department of Toxicology, Poznan University of Medical Sciences, Dojazd 30, 60-631 Poznań, Poland; kucinska@ump.edu.pl (M.K.); hpiotrow@ump.edu.pl (H.P.-K.); marek.murias@ump.edu.pl (M.M.); 4 Department of Molecular Biology and Translational Research, Institute of Rural Health, Jaczewskiego 2, 20-090 Lublin, Poland; lucynakapka@gmail.com; 5 World Institute for Family Health, Calisia University, 62-800 Kalisz, Poland

**Keywords:** silver nanoparticles, AgNPs, metastasis, cancer

## Abstract

The potential anticancer activity of different silver nanoformulations is increasingly recognized. In the present work, we use the model of 4T1 tumor in BALB/ccmdb immunocompetent mice to analyze the impact of citrate- and PEG-coated silver nanoparticles (AgNPs) on the development and metastatic potential of breast cancer. One group of mice was intragastrically administered with 1 mg/kg body weight (b.w.) of AgNPs daily from day 1 to day 14 after cancer cells implantation (total dose 14 mg/kg b.w.). The second group was intravenously administered twice with 1 or 5 mg/kg b.w. of AgNPs. A tendency for lowering tumor volume on day 21 (mean volumes 491.31, 428.88, and 386.83 mm^3^ for control, AgNPs-PEG, and AgNPs-citrate, respectively) and day 26 (mean volumes 903.20, 764.27, and 672.62 mm^3^ for control, AgNPs-PEG, and AgNPs-citrate, respectively) has been observed in mice treated intragastrically, but the effect did not reach the level of statistical significance. Interestingly, in mice treated intragastrically with citrate-coated AgNPs, the number of lung metastases was significantly lower, as compared to control mice (the mean number of metastases 18.89, 14.90, and 8.03 for control, AgNPs-PEG, and AgNPs-citrate, respectively). No effect of AgNPs treatment on the number of lung metastases was observed after intravenous administration (the mean number of metastases 12.44, 9.86, 12.88, 11.05, and 10.5 for control, AgNPs-PEG 1 mg/kg, AgNPs-PEG 5 mg/kg, AgNPs-citrate 1 mg/kg, and AgNPs-citrate 5 mg/kg, respectively). Surprisingly, inhibition of metastasis was not accompanied by changes in the expression of genes associated with epithelial–mesenchymal transition. Instead, changes in the expression of inflammation-related genes were observed. The presented results support the antitumor activity of AgNPs in vivo, but the effect was limited to the inhibition of metastasis. Moreover, our results clearly point to the importance of AgNPs coating and route of administration for its anticancer activity. Finally, our study supports the previous findings that antitumor AgNPs activity may depend on the activation of the immune system and not on the direct action of AgNPs on cancer cells.

## 1. Introduction

Silver nanoparticles (AgNPs) are among the most intensively studied and widely used categories of nanomaterials. This is mainly due to their antimicrobial activity, which makes them a valuable component of many cosmetic products, wound dressings, food packaging, and textiles. The mechanisms of AgNPs toxicity and their interaction with different aspects of cellular metabolism have been extensively studied during the last decade [1,2,3]. Several studies have also tested a potential anticancer activity of different AgNPs formulations. A majority of these studies are limited to in vitro models and, in most cases, report that AgNPs are cytotoxic to many cancer cell lines and inhibit their migration in vitro [4,5,6,7,8]. There are fewer studies analyzing AgNPs anticancer activity in animal models, but also reveal inhibition of tumor growth in vivo [9,10,11,12,13]. In vivo studies are mostly focused on tumor growth (tumor volume), whereas the AgNP effect on tumor metastatic potential is rarely studied. Nevertheless, Hu et al. report that Angstrom-scale silver particles inhibit both osteosarcoma growth and its metastatic potential in osteosarcoma-bearing nude mice [14]. Additionally, Kovacs et al. show that gold-core silver-shell nanoparticles inhibit tumor growth and metastatic dissemination of 4T1 tumors in mice [15]. Interestingly, this effect has been attributed to the modulation of a tumor-supporting activity of cancer-associated fibroblasts and not only to direct action of nanoparticles on cancer cells. Similarly, Chakraborty et al. and Manshian et al. observe that AgNPs anticancer activity in vivo depends on the triggering and/or enhancing of anticancer immune response [16,17]. This is in line with the immunomodulatory potential of AgNPs, which is observed in in vitro [18,19,20] and in vivo models [21,22,23]. This clearly shows that during the analysis of results of different studies concerning anticancer potential of nanoparticles, one must take into account not only the type, size, and coating of nanoparticles but also route of administration and presence of the fully functional immune system.

Cancer is a major public health problem worldwide and, according to cancer statistics, breast cancer is the most common cancer in women, with the second-highest mortality rate [24]. Ongoing research has enormous implications for improving the clinical outcome in breast cancer. This can be attributed to the progress made in the screening, diagnosis, and therapeutic strategies engaged in breast cancer management. Currently, there are several types of therapies available to treat breast cancer including hormone therapy, immunotherapy, and chemotherapy. Nevertheless, the resistance to therapy represents a substantial problem and generates a need for new therapeutic strategies. Among others, nanotechnology is expected to play important roles in future therapy for cancers, including metastatic breast cancer [25]. For that reason, in the present work, we use the model of 4T1 breast cancer tumor in BALB/ccmdb immunocompetent mice to analyze the impact of AgNPs on the development and metastatic potential of breast cancer, depending on nanoparticle coating and administration route.

## 2. Materials and Methods

### 2.1. Nanoparticles

Citrate-coated AgNPs (20 nm nominal size) were purchased from NanoComposix (San Diego, CA, USA). To obtain PEG-coated AgNPs, 1 mg of PEG-5000 (Sigma Aldrich, St. Louis, MO, USA) was dissolved in 100 µL of H_2_O and added to the 1 mL of 1 mg/mL citrate solution of AgNPs, and the reaction mixture was stirred for 2 h. For in vivo experiments, AgNPs were diluted in sterile water and administered to animals at a dose of 1 or 5 mg/kg. The in vitro and in vivo doses used in the present study were chosen based on data in the literature (for a recent review see [26]). Administration was performed intragastrically or intravenously through tail vein injection. Control animals received water. During in vitro experiments, AgNPs were vortexed for 30 s, and diluted in a cell culture medium without additional processing.

### 2.2. Animals

Female BALB/ccmdb mice (3 months of age) were obtained from the Center of Experimental Medicine, Medical University of Bialystok, Poland. Mice were kept on a standard laboratory diet (LaboFeed B from Morawski, Kcynia, Poland) with free access to drinking water. Animals were kept in community cages, under a 12 h day/night regime. Before the experiments, mice were quarantined and acclimatized for two weeks. All procedures were accepted by the 1st Local Ethics Committee for Experiments on Animals, permissions No. 109/2016, 215/2016, 216/2016, 217/2016.

### 2.3. 4T1 Breast Tumors

Mice were injected subcutaneously into the mammary fat pad with 1 × 10^5^ 4T1 cells suspended in 100 µL of PBS. Tumor growth was visible 5 days after inoculation. Tumor volume and the volume of blood vessels in tumor tissue were measured using the ultrasonographic imager Vevo 2100 (VisualSonics, Toronto, ON, Canada). Tumor volume (V) was estimated based on its three perpendicular diameters according to the formula: V = π/6 (a × b × c) [27]. During the animal section, metastases were counted using a binocular magnifier.

### 2.4. Cell Culture

The 4T1 mouse mammary gland carcinoma cell line was obtained from the American Type Culture Collection (ATCC). Cells were cultured in RPMI medium (Gibco, ThermoFisher Scientific, Waltham, MA, USA) supplemented with 10% fetal calf serum (Gibco, Thermo Fisher Scientific, Waltham, MA, USA). The cells were incubated in a 5% CO_2_ atmosphere at 37 °C.

### 2.5. RNA Isolation, Reverse Transcription and Real-Time PCR

The ReliaPrep RNA Cell Miniprep System (Promega, Madison, WI, USA) and ReliaPrep RNA Tissue Miniprep System (Promega, Madison, WI, USA) were used for RNA extraction from cells and tumor tissues, respectively. One microgram of total RNA was reverse-transcribed to cDNA in a 20 µL reaction volume using a High-Capacity cDNA Reverse Transcription Kit (Thermo Fisher Scientific, Waltham, MA, USA) according to the manufacturer’s instructions. cDNA was diluted to 150 µL with nuclease-free H_2_O. Real-time PCR was performed in a 20 µL reaction mixture containing 5 µL of cDNA, 4 µL of H_2_O, 10 µL of TaqMan Universal Master Mix II no UNG (Thermo Fisher Scientific, Waltham, MA, USA) and 1 µL of TaqMan Gene Expression Assay (Thermo Fisher Scientific, Waltham, MA, USA). The following TaqMan assays were used: Mm00495564_m1 (*Zeb1*), Mm00497193_m1 (*Zeb2*), Mm01247357_m1 (*Cdh1*), Mm01721878_m1 (*Pir*), Mm00441533_g1 (*Snai1*), Mm00441531_m1 (*Snai2*), Mm00502016_m1 (*Snai3*), Mm00440502_m1 (*Nos2*), Mm00443258_m1 (*Tnf*), Mm00434228_m1 (*Il1b*), Mm00434256_m1 (*Il2*), Mm00446190_m1 (*Il6*), Mm01288989_m1 (*Il12b*), Mm00518984_m1 (*Il23a*), Mm01288386_m1 (*Il10*), Mm01178820_m1 (*Tgfb1*), and Mm01197698_m1 (*Gusb*). PCR amplification was performed using a 7500 Real-Time PCR System (Thermo Fisher Scientific, Waltham, MA, USA) with an initial denaturation step for 10 min at 95 °C followed by 40 cycles of 95 °C for 15 s and 60 °C for 1 min. Gene expression was calculated using the ΔΔCt method, with *Gusb* as a reference control. Calculations were performed using Relative Quantification Software version 2019.2.7-Q2-19-build3 (Thermo Fisher Connect, www.thermofisher.com/pl/en/home/digital-science/thermo-fisher-connect.html, accessed on 16 April 2021, Thermo Fisher Scientific, Waltham, MA, USA).

### 2.6. The Wound Healing Assay

The 4T1 cells were seeded into 24-well plates (2.5 × 10^5^ cells/well) to grow in a monolayer for 24 h. Cells were incubated with AgNPs for two, four, or six hours and then cell culture medium containing nanoparticles was removed, and cells were washed three times with PBS. The cells were scratched with a pipette tip to generate two scratch wounds per well. The detached cells were removed by washing three times with PBS. Afterward, 1 mL of fresh cell culture medium was added to each well. Incubation and imaging were performed for 12 h using a motorized Nikon Eclipse Ti microscope (Nikon, Tokyo, Japan) equipped with a controlled environment chamber (Okolab, Pozzuoli, NA, Italy). The images of 2–3 fields within each well were captured automatically every one or two hours. The experiments were repeated three times. The cell migration rate (*Vmigration*) was calculated as described by Jonkman et al. [28]. Briefly, the gap area at each time point was determined using Image J software [29] and plotted against migration time. The *slope* of the resulting curve was used to calculate the cell migration rate from the following equation:

$$Vmigration=\frac{\left|slope\right|}{2\times l}$$

where *l* is the length of the gap.

### 2.7. Immunohistochemistry

Tumor samples were collected and cryopreserved in O.C.T and cut using Microm HM550 cryostat microtom (Thermo Fisher Scientific, Waltham, MA, USA). Sections (10 µm) were incubated for 5 min at room temperature, fixed in 100% cold acetone for 10 min, washed 2 times for 5 min with PBS, blocked with 1% BSA in PBS for 1 h, and again washed three times for 5 min with PBS. Sections were incubated with FITC-conjugated Cd11b monoclonal antibody (M1/70) (Invitrogen, Thermo Fisher Scientific, Waltham, MA, USA, Cat# 11-0112-82) at 4 µg/mL concentration for 4 h at room temperature. Subsequently, slides were washed 3 times for 5 min with PBS and counterstained with DAPI to identify nuclei. Immunofluorescence images were collected and analyzed using Nikon A1 confocal microscope (Nikon, Tokyo, Japan) equipped with NIS-elements AR 4.13.04 software (Nikon, Tokyo, Japan). Cd11b^+^ cell numbers per field were quantified in five 20× fields for each tumor tissue (*n* = 4 for control group and *n* = 3 for AgNPs-citrate group).

### 2.8. Statistical Evaluation

The statistical analysis was performed using Statistica 7.1 software (StatSoft, Tulsa, OK, USA). Statistical significances were evaluated using a t-test or ANOVA followed by Tukey’s post hoc test. Differences were considered statistically significant when *p* < 0.05.

## 3. Results

### 3.1. AgNPs Have No Effect on Tumor Growth, but Inhibit Its Metastatic Potential

To assess whether AgNPs can prevent tumor growth, one group of inoculated mice was intragastrically administered with 1 mg/kg body weight (b.w.) of AgNPs daily from day 1 to day 14 after cancer cells implantation (total dose 14 mg/kg b.w.). To evaluate whether AgNPs can attenuate tumor growth, the second group was intravenously administered twice with 1 or 5 mg/kg b.w. of AgNPs, with the first dose on day 5, when the primary tumor was visible and the second at midterm of tumor growth (day 14), a total dose of 2 or 10 mg/kg b.w. Control animals received water. During the pilot experiment, metastases began to appear on the surface of the lungs 20–25 days after tumor implantation. The number of metastases increased markedly over the next five days, leading to a deterioration of the animal’s condition approximately 30 days after inoculation. To minimize suffering, in the main experiment, the animals were sacrificed and their tissues were harvested for analysis on day 26. Two types of AgNPs were used: citrate- and PEG-coated AgNPs, both with a nominal size of 20 nm. Due to the high toxicity of citrate-coated AgNPs observed in the pilot experiment after the second intravenous administration at a dose of 5 mg/kg b.w, in the main experiment, this dose was administered intravenously only once, on day 5.

**Figure 1:**
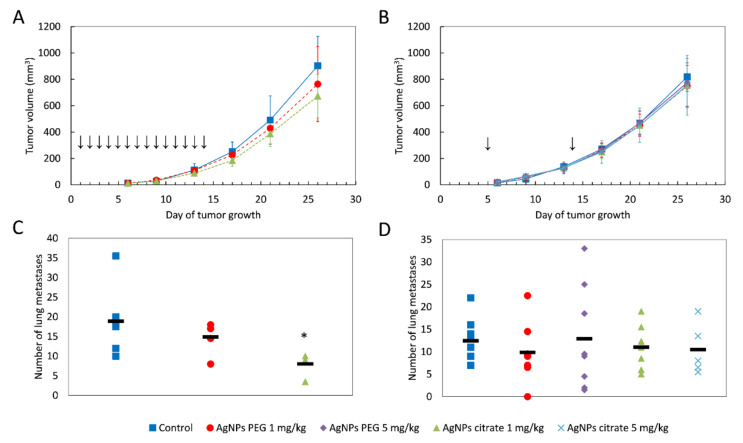
Tumor volume and the number of lung metastases in mice treated intragastrically (**A**,**C**) or intravenously (**B**,**D**) with citrate- or PEG-coated AgNPs. In A and B, data are presented as the mean ± standard deviation and arrows indicate the day of AgNPs administration. In C and D, the black rectangle represents the mean and the markers represent data from individual mice. * *p* < 0.05, *n* = 6.

**Figure 2:**
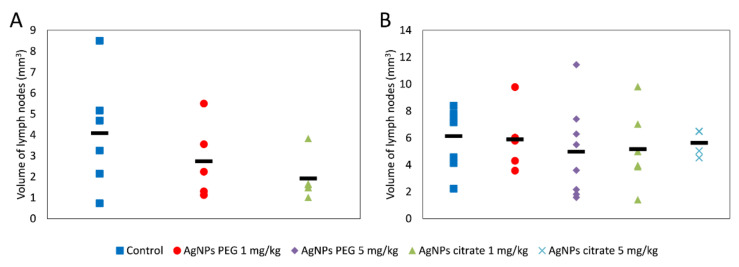
The summed volume of axillary and inguinal lymph nodes located on the same side of the body as the tumor in mice treated intragastrically (**A**) or intravenously (**B**) with citrate- or PEG-coated AgNPs. The black rectangle represents the mean and the markers represent data from individual mice. The differences are not statistically significant, *n* = 6.

A tendency for lowering tumor volume on days 21 and 26 has been observed in mice treated intragastrically, but the effect did not reach the level of statistical significance (Figure 1A) and was much less visible after intravenous AgNPs administration (Figure 1B). Interestingly, in mice treated intragastrically with citrate-coated AgNPs, the number of lung metastases was significantly lower, as compared to control mice (Figure 1C). A similar, but less pronounced and not statistically significant effect was observed for PEG-coated AgNPs. No effect of AgNPs treatment on the number of lung metastases was observed after intravenous administration (Figure 1D). A similar tendency was observed for the volume of axillary and inguinal lymph nodes located on the same (right) side of the body as the tumor. After intragastrical administration, lymph node volume was the lowest for citrate-coated AgNPs (Figure 2A). After intravenous administration, differences in right-sided lymph node volume were negligible (Figure 2B). Volumes of axillary and inguinal lymph nodes located on the left side of the body were unaffected by AgNPs treatment (data not shown).

### 3.2. AgNPs Have a Negligible Effect on the Volume of Blood Vessels in Tumor Tissue

**Figure 3:**
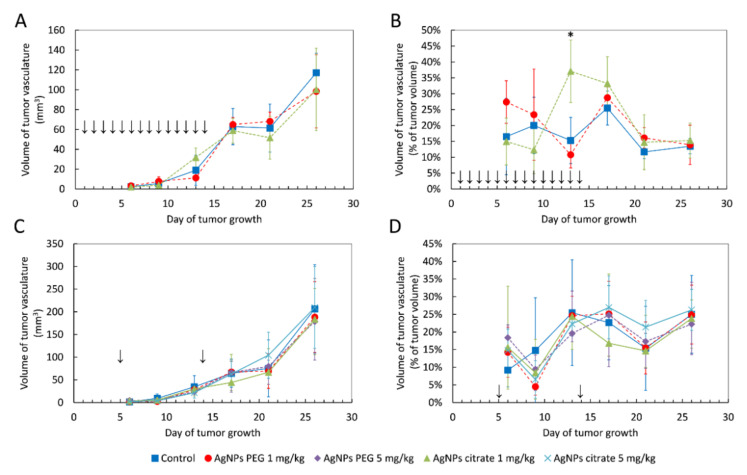
The volume of blood vessels in tumor tissue shown as an absolute value or as a percent of tumor volume in mice treated intragastrically (**A**,**B**) or intravenously (**C**,**D**) with citrate- and PEG-coated AgNPs. Arrows indicate AgNPs administration. Data are presented as the mean ± standard deviation, *n* = 6. * *p* < 0.05.

Using the same in vivo model of breast cancer as in the present work, we recently showed that gold nanoparticles transiently increased the volume of blood vessels in tumor tissue after both intragastrical and intravenous administration [30]. In order to check if a similar effect can be induced by AgNPs, we performed an analysis of the volume of blood vessels in tumor tissue after AgNPs treatment. In contrast to gold nanoparticles, AgNPs had a negligible effect on the volume of blood vessels. A significant increase in the volume of tumor vasculature was observed only on day 13 after tumor implantation in the case of intragastric administration of citrate-coated AgNPs (Figure 3A,B). No such effect was observed for intravenous AgNPs administration (Figure 3C,D).

### 3.3. AgNPs Have No Effect on the Expression of Genes Associated with Epithelial–Mesenchymal Transition In Vivo and Migration of 4T1 Cells In Vitro

**Figure 4:**
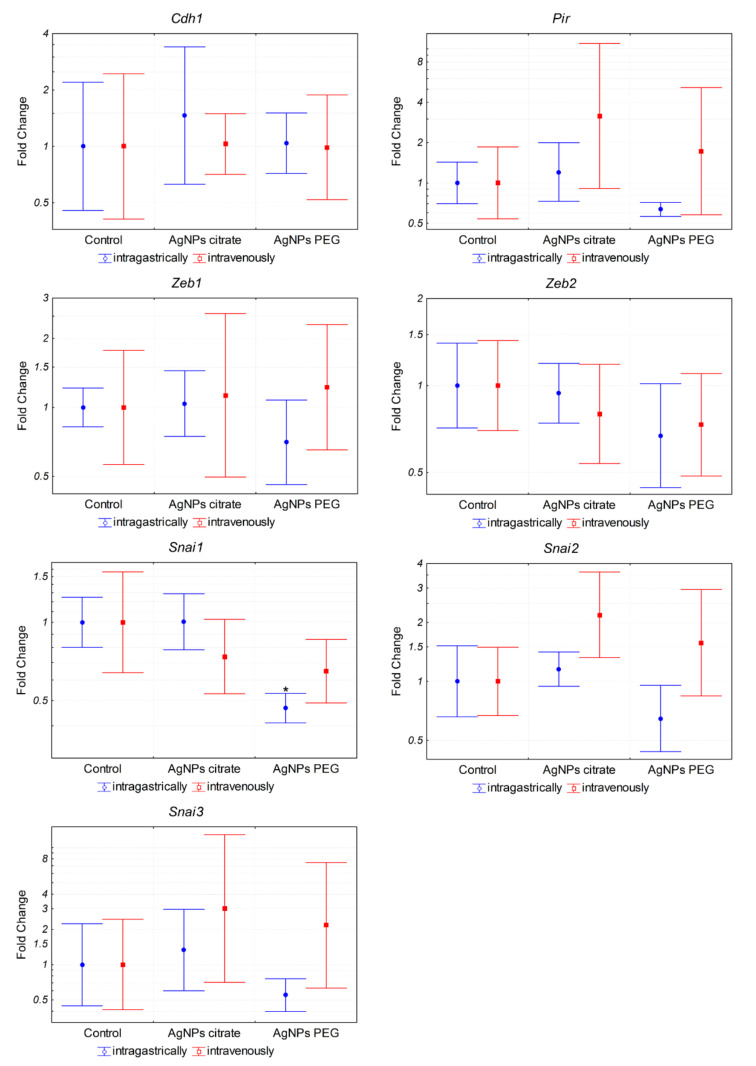
Expression of EMT-related genes at the mRNA level in tumors from mice treated intragastrically or intravenously with 1 mg/kg citrate- or PEG-coated AgNPs. Markers represent mean fold change values and whiskers represent the minimum and maximum fold change calculated from a standard deviation of ΔCt values. * *p* < 0.05 difference versus control group, *n* = 6.

**Figure 5:**
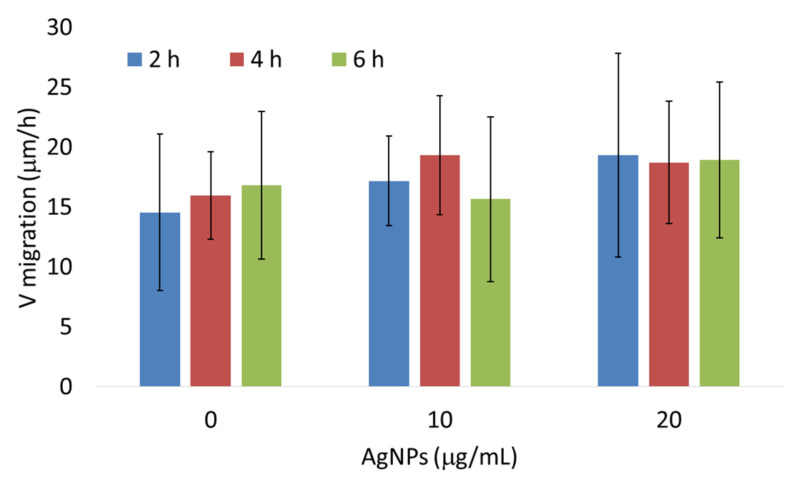
Citrate-coated AgNPs had no effect on 4T1 cell migration in vitro as measured by the wound healing assay. The data are presented as means and standard deviations from three independent experiments. The differences are not statistically significant.

In order to elucidate the mechanism of metastasis inhibition by citrate-coated AgNPs, we analyzed the expression of genes associated with epithelial–mesenchymal transition (EMT) and metastasis in tumor samples from mice treated intragastrically with AgNPs. Analyzed genes included transcription factors *Zeb1*, *Zeb2*, *Snai1*, *Snai2*, and *Snai3* and genes encoding cadherin 1 (*Cdh1*) and pirin (*Pir*) [31,32,33]. The only statistically significant change was a ~2-fold decrease in Snai1 expression in mice treated with PEG-coated AgNPs (Figure 4). It is well established that Snai1 transcription factor overexpression is associated with increased tumor migration and invasion via induction of EMT [31]. Therefore, it could be expected that the observed decrease in lung metastases after intragastrical administration of citrate-coated AgNPs would be associated with a decrease in *Snai1* expression. Instead, a decrease in *Snai1* expression was observed in mice treated with PEG-coated AgNPs for which there was no significant change in the number of lung metastases. Such a result suggests that the observed decrease in lung metastases after intragastrical administration of citrate-coated AgNPs was not due to the direct action of AgNPs on tumor cells and the decrease in their EMT potential. To further test this hypothesis, we analyzed the impact of citrate-coated AgNPs on the migration of 4T1 cells in vitro by means of the wound healing assay. No effect of AgNPs on the migration of 4T1 cells in vitro was observed, as shown in Figure 5.

### 3.4. AgNPs Induce Expression of Pro-Inflammatory Genes in Tumor Cells

**Figure 6:**
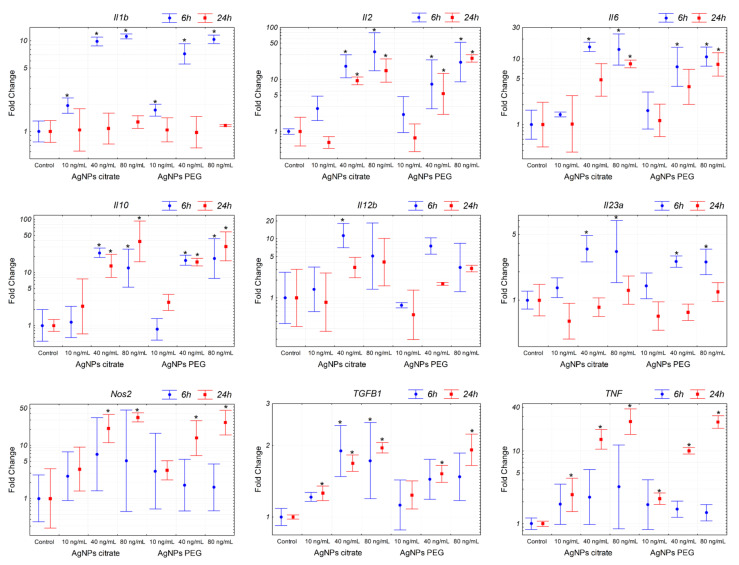
Expression of genes related to inflammation in 4T1 cells treated in vitro with citrate- or PEG-coated AgNPs for 6 or 24 h. Markers represent mean fold change values and whiskers represent the minimum and maximum fold change calculated from a standard deviation of ΔCt values. * *p* < 0.05 difference versus control group, *n* = 3.

**Figure 7:**
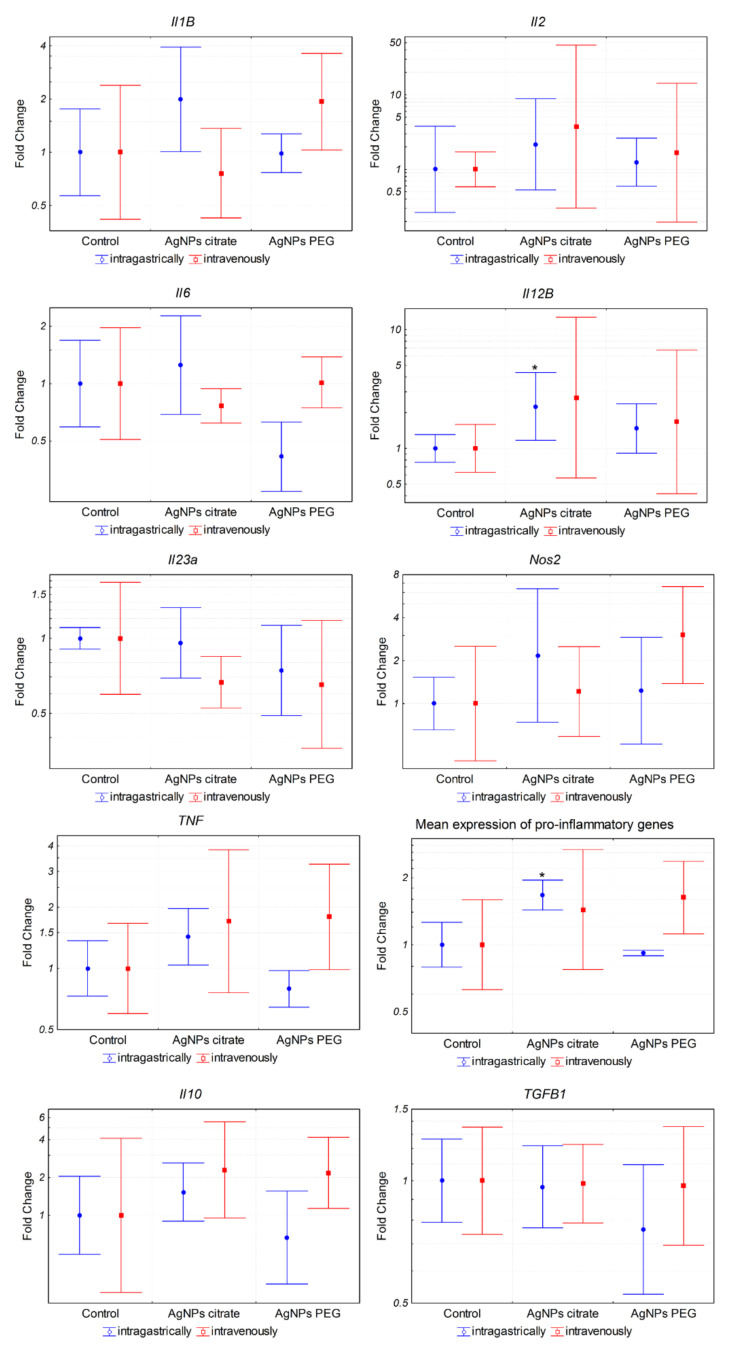
Expression of inflammation-related genes in tumor tissue from mice treated intragastrically or intravenously with 1 mg/kg citrate- or PEG-coated AgNPs. Markers represent mean fold change values and whiskers represent the minimum and maximum fold change calculated from a standard deviation of ΔCt values. * *p* < 0.05 difference versus control group, *n* = 6.

The results described above suggested that the observed decrease in the number of lung metastases following intragastric administration of citrate-coated AgNPs was not related to the decreased metastatic potential of tumor cells per se. We hypothesized that the effect was due to the activation of the immune system triggered by AgNPs, thus we decided to analyze the expression of inflammation-related genes in 4T1 cells. The analysis revealed a significant induction of pro-inflammatory (*Il1b*, *Il2*, *Il6*, *Il12b*, *Il23a*, *Tnf*, and *Nos2*) and anti-inflammatory (*Il10* and *Tgfb1*) genes after in vitro incubation with citrate- or PEG-coated AgNPs (Figure 6). Interestingly, both types of AgNPs induced similar changes in gene expression. This result indicated that AgNPs had the potential to induce the expression of genes, which may modulate the function of the immune system. In the next step, we checked if the expression of these genes was also affected in tumor tissue in vivo. In this case, a significant increase was observed only for Il12b after intragastrical administration of citrate-coated AgNPs (Figure 7). Interestingly, despite no other genes showing statistically significant changes, when we analyzed the mean expression of pro-inflammatory genes calculated from the mean ΔCt value of these genes, it was almost 2-fold higher in tumor tissue from mice treated intragastrically with citrate-coated AgNPs than in matched control (Figure 7). The fact that changes in gene expression observed in vivo were much smaller compared to in vitro experiments may be explained by the fact that tumor samples were collected at the end of the experiment, i.e., on the 26th day of tumor growth, while the last AgNPs administration was performed on day 14. It is possible that higher changes would be observed at earlier time points.

### 3.5. Citrate-Coated AgNPs Have No Effect on the Number of Cd11b^+^ Cells Infiltrating the Tumor

**Figure 8:**
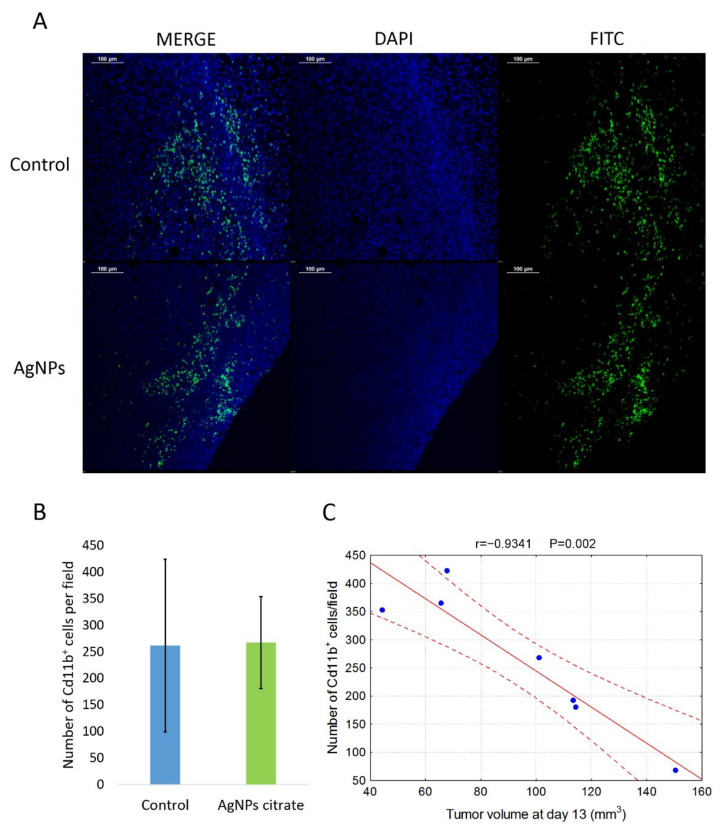
(**A**) Representative images of Cd11b^+^ cells localization in tumor tissue from control and citrate-AgNPs-treated mice. Cell nuclei were stained with DAPI and Cd11b^+^ cells were stained with FITC-conjugated antibody. (**B**) Mean number ± standard deviation of Cd11b^+^ cells per field in tumor tissue from four control mice and three AgNPs-treated mice. (**C**) Correlation between the number of Cd11b^+^ cells and tumor volume on day 13 of tumor growth.

To further clarify the mechanism of AgNPs-mediated metastasis inhibition, we compared the number of Cd11b^+^ cells in tumor tissue from control mice and mice treated intragastrically with citrate-coated AgNPs. Immunohistochemistry analysis shows no differences between groups under study in the number of Cd11b^+^ cells infiltrating tumor tissue (Figure 8A,B). Interestingly and in agreement with data in the literature [34], there was a strong negative correlation between the presence of Cd11b^+^ cells and tumor volume, especially on day 13 of tumor growth (Figure 8C).

## 4. Discussion

The antimicrobial activity of AgNPs is the main reason for their wide use as an additive to many consumer products. However, the potential anticancer activity of different silver nanoformulations is increasingly recognized and the number of studies on this topic is increasing. So far, it is difficult to draw definite conclusions from these studies given that different AgNPs formulations are used on different cancer models with different dosing and administration schemes. In particular, it should be recognized that in most of the studies involving testing of AgNPs anticancer activity in vivo, biologically synthesized, “biogenic” AgNPs are used [9,10,11,12,13,35]. Biological synthesis involves the use of extracts from different organisms, such as bacteria, fungi, algae, or plants, and obtained nanoparticles are coated with proteins and compounds derived from these extracts. Therefore, the observed anticancer effects may be, at least partially, due to compounds from biological extracts and not due to AgNPs per se. In the present study, we used non-biogenic AgNPs coated with citrate or PEG and no significant impact on tumor implantation or growth has been observed (Figure 1A,B). This inconsistency with other reports is probably due to differences in AgNPs formulation or administration route. In in vivo studies published to date, AgNPs were administered peritumorally [15,17], subcutaneously [16], intraperitoneally [10,11,13], intravesically [9], or intravenously [14]. To the best of our knowledge, the present study is the first report on anticancer AgNPs activity after intragastrical administration.

In the present study, the anticancer activity of AgNPs was manifested by inhibition of metastasis formation (Figure 1C) and by a statistically insignificant trend toward inhibition of tumor growth (Figure 1A). This activity was clearly dependent on nanoparticle coating (the effect was observed only for citrate-coated AgNPs) and administration route (the effect was observed only after intragastrical administration). In vivo distribution of AgNPs is dependent on various mechanisms, such as opsonization, uptake by the mononuclear phagocyte system, protein corona formation, an enhanced permeability and retention effect, and lymphatic transport [36]. It is possible that observed differences in the anticancer activity of citrate- and PEG-coated AgNPs are due to differences in absorption and/or distribution arising from different coating materials. Moreover, to some extent, the observed effects may be due to silver ions released from the nanoparticles, and the rate of ion release from citrate- and PEG-coated AgNPs may differ. It should also be noted that the total doses and dosing regimens differed between intragastric and intravenous administration, which could have an impact on the final results. Similar effects on metastasis formation were recently observed by Hu et al. for fructose-coated Angstrom-scale silver particles administered intravenously [14] and by Kovacs et al. for gold-core silver-shell nanoparticles administered peritumorally [15].

**Figure 9:**
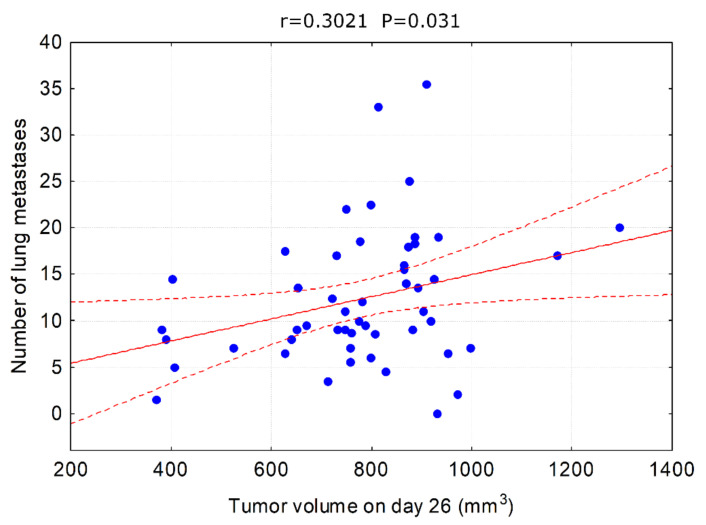
Correlation between tumor volume on day 26 and the number of lung metastases. All animals used in the experiment are analyzed together.

One can argue that the observed inhibition of metastasis formation is due to slower (although not in terms of statistical significance) tumor growth and hence delayed start of the metastasis process. However, correlations between tumor size and metastases can be misleading as metastatic spreading is not the exclusive hallmark of the late tumor development but the invasive cells can appear at the very beginning of the cancerous transformation to form distant lesions. If the tumor growth rate was the main factor influencing the formation of metastases, then we should observe a strong correlation between tumor volume and the number of metastases. This was not the case in our experimental model. A statistically significant correlation between tumor volume and the number of metastases was observed only on day 26 and it was weak (Figure 9), suggesting that other mechanisms were involved.

Metastasis is a complex process consisting of two main stages. In the first stage, cancer cells migrate from the primary tumor into the surrounding tissues and vasculature of the lymph and blood systems. In the second step, cells exit the bloodstream, invade new tissues, and form secondary tumors. Invasive and migratory capabilities of cancer cells require so-called epithelial–mesenchymal transition (EMT), which is a cellular process that converts immotile epithelial cells to motile mesenchymal cells by disrupting cell adhesion. A switch in gene expression from epithelial to mesenchymal phenotype is triggered by complex regulatory networks involving transcriptional control with *Snai1*, *Snai2*, *Snai3*, *Zeb1*, and *Zeb2* transcription factors that regulate the expression of genes involved in EMT, such as *Cdh1* or *Pir* [31,32]. Surprisingly, in our experimental setup, inhibition of metastasis was not accompanied by changes in the expression of EMT-related genes in tumor tissue (Figure 4), which suggests that the effect was not caused by the direct action of AgNPs on cancer cells. This was confirmed by an in vitro experiment in which AgNPs had no effect on 4T1 cells migration (Figure 5).

Several studies based both on in vitro and in vivo experiments reported that AgNPs had an immunomodulatory potential. Yang et al. showed the therapeutic potential of PEGylated and modified (with folic acid) AgNPs for rheumatoid arthritis and demonstrated that they were highly effective for alleviating inflammation via modulation of macrophages polarization [21]. Effects of AgNPs on the immune system were also observed in vivo after intravenous administration in rats [22,23]. Poon et al. showed that AgNPs induced expression of genes involved in several innate and adaptive immunity-associated pathways in THP-1 derived macrophages [19]. Similarly, Murphy et al. reported that AgNPs exposure resulted in upregulation of inflammatory cytokines IL-1, IL-6, and TNF in THP-1 cells and primary human monocytes, which was accompanied by inflammasome formation. The authors concluded that AgNPs exposure can result in an innate immune response and may potentially contribute to the risk of disease development or indeed exacerbate already existing conditions by inducing an immunologically active state [18]. Chakraborty et al. reported that mouse serum albumin-coated AgNPs inhibited tumor growth and attributed the effect to the activity of the immune system [16]. In subsequent work, they showed in vitro that AgNPs-induced oxidative stress triggers tumor-associated macrophages (TAMs) reprogramming from M2 anti-inflammatory to M1 pro-inflammatory phenotype [37,38]. In line with this, peritumoral administration of citrate-coated AgNPs to immune competent mice induced inflammation, resulting in a significantly reduced tumor growth, as compared to AgNP-treated tumors in an immune-deficient mouse model [17]. To check if in our experimental model AgNPs have the potential to induce inflammation or trigger an antitumor immune response, we analyzed the expression of pro and anti-inflammatory genes in 4T1 cells after AgNPs treatment in vitro. Indeed, significant changes were observed, confirming the immunomodulatory potential of AgNPs (Figure 6). Interestingly, results obtained for citrate- and PEG-coated AgNPs were similar, which implied that different actions of these nanoparticles observed in vivo could not be reduced only to interaction with cancer cells and therefore could not be easily recapitulated in vitro. This is in line with the analysis of the expression of inflammation-related genes in vivo in tumor tissue where a significant effect was observed for citrate-coated but not PEG-coated AgNPs (Figure 7).

Among the types of immune cells that play important roles in the interaction between tumor and immune system are tumor-associated macrophages, which can have a pro-tumorigenic (so-called M2) phenotype or antitumorigenic (M1) phenotype. Importantly, the presence and phenotype of TAMs affect not only the growth of primary tumors but also the development of metastases [39]. Recently, Schmid et al. showed that integrin Cd11b is crucial for the antitumor activity of TAMs [34]. In line with their results, we observed a negative correlation between tumor volume and the presence of Cd11b^+^ cells in tumor tissue (Figure 8C). Nevertheless, we did not observe any difference between the number of Cd11b^+^ cells in tumor tissue from control mice and citrate-AgNPs-treated mice (Figure 8A,B).

It should be emphasized that in our experiments, tumor samples were collected on day 26 after tumor implantation, while the last AgNPs dose was administrated on day 14. Therefore, it is possible that parameters, such as gene expression and Cd11b^+^ cells infiltration, already returned to the basal level, even if they were transiently affected by AgNPs treatment.

Since silver is a well-known antimicrobial agent, its intragastric administration can cause significant changes in the gut microbiome [40]. Moreover, many studies demonstrated the impact of the gut microbiome on tumor growth and dynamics, with effects on the immune system as among possible mechanisms [41,42]. Consequently, it can be hypothesized that the mechanism of metastasis inhibition after intragastric AgNPs administration observed in the present work depended on AgNPs-induced gut microbiome changes. This issue is beyond the scope of the present study but is definitely worth investigating in the future.

Another interesting aspect analyzed during the present study was the volume of blood vessels in tumor tissue after AgNPs administration. In our recent work using the same experimental model, we showed that gold nanoparticles induce transient vasodilation in tumor tissue after both intragastric and intravenous administration [30]. In the present study, we did not observe such an effect, which proved that the vasodilating effect was specific to gold nanoparticles.

## 5. Conclusions

Results of the present work support the antitumor activity of AgNPs in vivo, but the effect was limited to the inhibition of metastasis, while the growth of the primary tumor was unaffected. The novelty of this work lies in the fact that it clearly points to the importance of AgNPs coating and route of administration for its anticancer activity. Moreover, our study supports previous findings that antitumor AgNPs activity may depend on the activation of the immune system and not on the direct action of AgNPs on cancer cells. The mechanistic aspects of AgNPs-triggered antitumor immune system activation remain to be fully elucidated but the results of the present work may help to develop new, efficient, nanoparticle-based anticancer therapies.

## Data Availability

The raw data are available upon request from the corresponding author.
